# Utilizing Clinical and Non-clinical Patient Factors in Predicting Cardiovascular Events in Patients on JAK Inhibitor Therapy: A Retrospective Cohort Study

**DOI:** 10.7759/cureus.48595

**Published:** 2023-11-10

**Authors:** Kehinde O Sunmboye, Tom Petrie, Billy Bui, Hassan Salim, Mutal Khan

**Affiliations:** 1 Rheumatology, College of Health Sciences, University of Leicester, Leicester, GBR; 2 Rheumatology, University Hospitals of Leicester NHS Trust, Leicester, GBR; 3 Rheumatology, University of Leicester, Leicester, GBR

**Keywords:** social deprivation, cardiovascular prevention strategy for cardiovascular events, psoriatic arthritis, • rheumatoid arthritis, jak inhibitors

## Abstract

Background: Patients with autoimmune rheumatic diseases (ARDs) taking JAK inhibitors may have an increased risk of cardiovascular events, especially if they have other health conditions. Identifying high-risk patients can inform targeted preventive care. This study assessed the value of age and deprivation decile in predicting cardiovascular events in patients on JAK inhibitors for ARDs.

Objective: To assess the predictive value of age and deprivation decile in identifying patients at risk of cardiovascular events while on JAK inhibitor therapy for ARDs.

Methods: This cross-sectional cohort study enrolled 309 patients with ARDs (mean age 59.3 years, 77% female) treated with JAK inhibitors at a UK teaching hospital. Baseline characteristics, including age, gender, ethnicity, and comorbidities, were collected. Cardiovascular events (myocardial infarctions, strokes, and cardiovascular-related deaths) that occurred while on JAK inhibitor therapy were identified retrospectively. Deprivation indices were calculated using socioeconomic factors.

Results: Multivariate logistic regression analysis, adjusting for potential confounders, showed that a model combining age and deprivation decile was statistically significant (p = 0.031) in predicting cardiovascular events. Neither age nor deprivation decile alone was statistically significant. Older patients had an odds ratio of 1.06 (95% CI: 1.00-1.13) for increased risk of cardiovascular events. The logistic regression model as a whole was statistically significant (Chi2(14) = 24.04, p = 0.031, n = 309). The AUC of the ROC curve was 0.837.

Conclusion: Age and deprivation decile can effectively predict cardiovascular events in patients on JAK inhibitor therapy for ARDs. Incorporating these predictive tools into routine clinical practice can help identify patients who warrant intensified cardiovascular risk management.

## Introduction

Cardiovascular disease remains one of the leading causes of morbidity and mortality worldwide [1]. Patients with autoimmune rheumatic diseases, such as rheumatoid arthritis, psoriatic arthritis, and ankylosing spondylitis, are at an increased risk of cardiovascular events compared to the general population [2]. In recent years, the introduction of Janus kinase (JAK) inhibitor therapy has provided effective therapeutic options for managing these rheumatic conditions, offering improved disease control and better quality of life for patients [3,4].

However, the use of JAK inhibitor therapy has prompted concerns regarding their potential cardiovascular adverse effects in certain high-risk patients with multiple co-morbidities [5,6]. Several studies have explored the association between JAK inhibitor therapy and cardiovascular risk in patients with autoimmune rheumatic diseases [6], but the results have been inconclusive or limited to evaluating the co-morbid patient factors that may increase cardiovascular risk in these patients. Therefore, a comprehensive evaluation of the predictive value of patient factors and non-patient factors to determine cardiovascular risks associated with JAK inhibitor therapy in a targeted patient population is warranted.

In addition to clinical patient factors, other non-clinical patient factors like socio-economic factors such as deprivation deciles have also been evaluated as a tool to help predict the likelihood of developing cardiovascular events [7]. Deprivation deciles have proven to be useful indicators in predicting cardiovascular events. By categorizing individuals into various deprivation levels based on their socioeconomic status, these deciles serve as a reflection of the degree of social disadvantage or advantage they experience (1=most deprived, 10=least deprived).

Studies have shown that individuals in higher deprivation deciles are more likely to have poorer health outcomes, including a higher risk of developing cardiovascular diseases [8,9]. This association can be attributed to several factors, such as limited access to healthcare services, unhealthy lifestyle choices, and increased exposure to various risk factors. Therefore, by considering an individual's deprivation decile, healthcare professionals can better identify those at higher risk of cardiovascular events and provide targeted interventions and preventive measures to mitigate these risks.

In addition to this, the age of patients has always been implicated as an independent risk factor for cardiovascular events in the older patient groups [10]. The occurrence of cardiovascular disease (CVD) has been observed to rise as individuals grow older, affecting both males and females. This includes an increased prevalence in the occurrence of conditions such as atherosclerosis, stroke, and myocardial infarction [10].

Older patients on biologic therapy for autoimmune rheumatic disease have also been thought to be at increased risk of adverse outcomes compared to age-matched individuals not on biologic therapy [11]. However, studies have indicated that there is a need for more robust systematic studies and meta-analyses to quantify this risk in older patients on biologic therapy [11]. A large prospective national cohort study of 4140 patients actually showed a reduction in cardiovascular events in older patients on biologic therapy suggesting that increasing age alone is not an independent risk factor for increased cardiovascular events whilst on biologic therapy [12]. Targeted synthetic medication (JAK inhibitor therapy) is a subset of biologic therapies for treating autoimmune rheumatic diseases that have been suggested in studies to cause increased cardiovascular events although this remains an area of controversy as results from various studies have been mixed [13].

To address the existing gap in knowledge, the present study aims to investigate the potential risk factors associated with cardiovascular events in patients receiving JAK inhibitor therapy. Previous research on this topic has yielded inconsistent findings, particularly concerning the impact of age and drug-related factors. Therefore, a cross-sectional cohort study was undertaken by the authors to examine these variables and shed light on their potential influence on cardiovascular event occurrence.

The association between patient factors (age, gender, ethnicity, rheumatic disease subtype, comorbidities) and type of JAK inhibitor therapy with cardiovascular events was investigated. By leveraging electronic health records and utilizing the health records of patients registered on the biologics database, the authors were able to assess the impact of patient factors on cardiovascular risks and provide valuable insights for clinical decision-making in prescribing JAK inhibitor therapy.

## Materials and methods

Study design and setting

The authors conducted a cross-sectional retrospective cohort study of patients with rheumatoid arthritis (RA), psoriatic arthritis (PsA), or ankylosing spondylitis (AS) who were prescribed JAK inhibitor therapy at three hospital sites belonging to one NHS University Teaching Hospital in the United Kingdom. The study period was May-July 2023.

Participants, data source, and collection

Data were extracted from the biologics database, which is a comprehensive database of patients with rheumatic diseases receiving biologics treatment. The database contains data on individual patient baseline characteristics, including age, gender, ethnicity, diagnosis, family history of cardiovascular disease, lipid profiles, and other patient comorbidities. Q-risk (QRISK®3) scores and deprivation deciles were also determined for the identified patients.

Patients were identified for the study using a standardized search algorithm. The search algorithm identified patients with rheumatic diseases who were receiving JAK inhibitor therapy and who had at least 12 months of follow-up data. The identified patients were then assessed for any recorded cardiovascular events (such as angina, myocardial infarction, stroke, and cardiovascular-related deaths, based on standardized diagnostic codes) while on JAK inhibitor therapy.

Patient consent to use the data on the biologics database was obtained prior to enrolment on the biologics database. The data was used in line with the hospital research ethics guidelines.

Eligibility criteria

Inclusion Criteria

All patients over 18 years old with a diagnosis of rheumatoid arthritis, psoriatic arthritis, and ankylosing spondylitis on JAK inhibitor therapy at the time of the study were included in the data collection and final analysis.

Exclusion Criteria

Patients with a history of cardiovascular disease were excluded from the study. None were encountered in the study cohort.

Outcomes and exposures

All patients who had cardiovascular events (angina, myocardial infarction, stroke, or cardiovascular-related death) while on JAK inhibitor therapy.

Independent Variables (Predictors and Potential Confounders)

Independent variables include age, gender, ethnicity, diagnosis (rheumatoid arthritis, psoriatic arthritis, ankylosing spondylitis), family history of cardiovascular disease, lipid profiles, other patient comorbidities (hypertension, dyslipidemia, diabetes), calculated Q-risk (QRISK®3) score, and the deprivation decile. There were no effect modifiers identified.

Outcome Definition

The primary outcome of the study was the occurrence of any cardiovascular event (angina, myocardial infarction, stroke, or cardiovascular-related death), dichotomized as yes or no.

Cardiovascular Event Identification

Patients who had any cardiovascular events, including angina, myocardial infarction, stroke, and cardiovascular-related deaths, were identified based on standardized diagnostic codes. Angina was defined using ICD-10 code 120, myocardial infarction was defined using ICD-10 code I21, and stroke was defined using ICD-10 code I63. Cardiovascular-related death was defined using ICD-10 codes I11, I13, I20-I25, I27-I28, and I50.

Statistical analysis

All statistical analyses were performed using DATAtab® (DATAtab: Online Statistics Calculator. DATAtab e.U. Graz, Austria), an online statistical software analysis tool. A multivariate logistic regression analysis was used to assess the association between all the predictor variables and the dichotomous outcome of cardiovascular events. The regression analysis model was adjusted for all potential confounding factors. Various independent variables (such as age, deprivation deciles, gender, ethnicity, disease subtype, etc.) were assessed along with cardiovascular events, both individually and in combination. Statistical significance was determined using p-values, with a p-value < 0.05 considered statistically significant.

Model Development and Evaluation

A multivariate logistic regression model was developed using a backward selection approach. All selected covariates were initially included in the model, and then they were removed one by one until only the statistically significant covariates remained. The model fit was assessed using the Pearson chi-squared test and the area under the receiver operating characteristic curve (AUC).

Quantitative Variables and Covariate Selection

Quantitative variables, such as age and Q-risk (QRISK®3, University of Nottingham Nottingham, UK; EMIS Group, Leeds, UK) score, were handled in the analyses as continuous variables. Covariates were selected for the multivariate logistic regression model based on their potential confounding influence on the relationship between independent variables and cardiovascular events.

Bias

Several steps were taken to address potential sources of bias. First, the study was designed as a cross-sectional retrospective cohort study, which reduces the risk of selection bias. Second, all eligible patients were included in the study, regardless of their exposure to JAK inhibitor therapy. Third, the study used a variety of variables to control for potential confounding factors. Fourth, the study was conducted in a large and diverse population, which reduces the risk of generalizability bias.

Additional Methods

Q-risk (QRISK®3) score calculation: the Q-risk (QRISK®3) score as a measure of cardiovascular risk was calculated using the online Q-risk (QRISK®3) calculator at https://qrisk.org/

Deprivation decile calculation: the deprivation decile as a measure of socioeconomic status was calculated using the online English Index of Multiple Deprivation (IMD) postcode checker at https://www.fscbiodiversity.uk/imd/

Sensitivity Analysis

In a sensitivity analysis, we excluded patients with a history of cardiovascular disease prior to starting JAK inhibitor therapy and then evaluated the association between individual and combined patient factors and cardiovascular events in the study cohort.

## Results

Descriptive statistics

A total of 309 patients with a mean age of 59 years (SD +/- 12.3 years). The majority were female (77%). Table 1 shows that 73% of the study cohort were Caucasian, with 25% of South Asian ethnic origin. Only 0.6% were of Black ethnic origin. Table 1 also provides the demographic characteristics of the mean age of study participants.

**Table 1:** Patient mean age, ethnicity with minimum and maximum age

Ethnicity	Frequency	Percentage %	Mean age (years)	Std. Deviation	Minimum age (years)	Maximum age (years)
White	227	73.46%	60.04	12.35	19	87
South Asian	79	25.57%	57.61	12.41	20	87
Black	2	0.65%	55.5	0.71	55	56
White (Any Other)	1	0.32%	50	N/A	50	50

**Figure 1:**
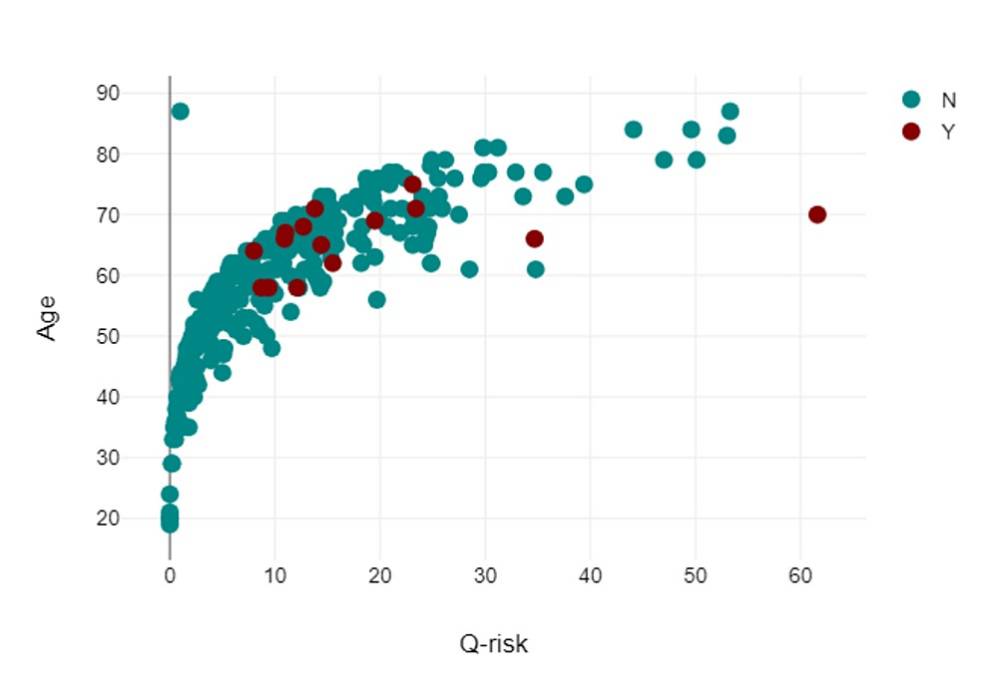
Scatter diagram showing Q-risk (QRISK®3) value and cardiovascular event by age

Figure 1 shows via scatter diagram, the distribution of the Q-risk by age. The median Q-risk (QRISK®3) score was 11.54% (range: 0-62%), indicating a moderate cardiovascular risk.

Table 2 gives a 2 X 2 representation of patients with cardiovascular events on the database. Out of the 309 patients observed, there was no missing data so all 309 patients were used in the final analysis. 

**Table 2:** 2 X 2 table showing the records of cardiovascular events in patients on biologics database on JAK inhibitor therapy

	Yes	No
Predicted events	0	309
Observed events	14	295

**Figure 2:**
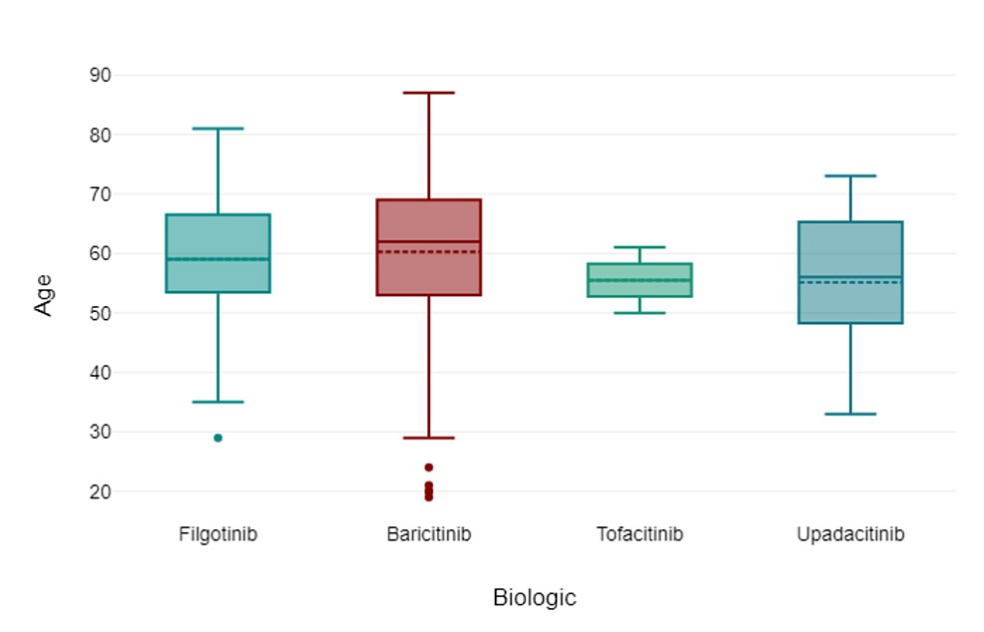
Age of patient by JAK inhibitor therapy used

**Figure 3:**
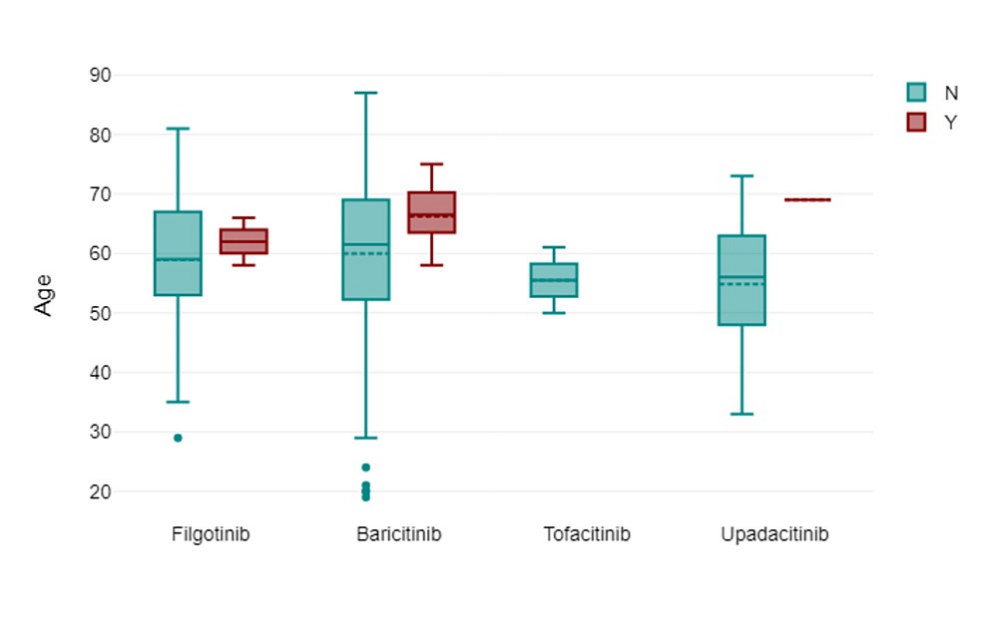
Box plot showing Incidence of cardiovascular events by age and JAK inhibitor therapy

**Figure 4:**
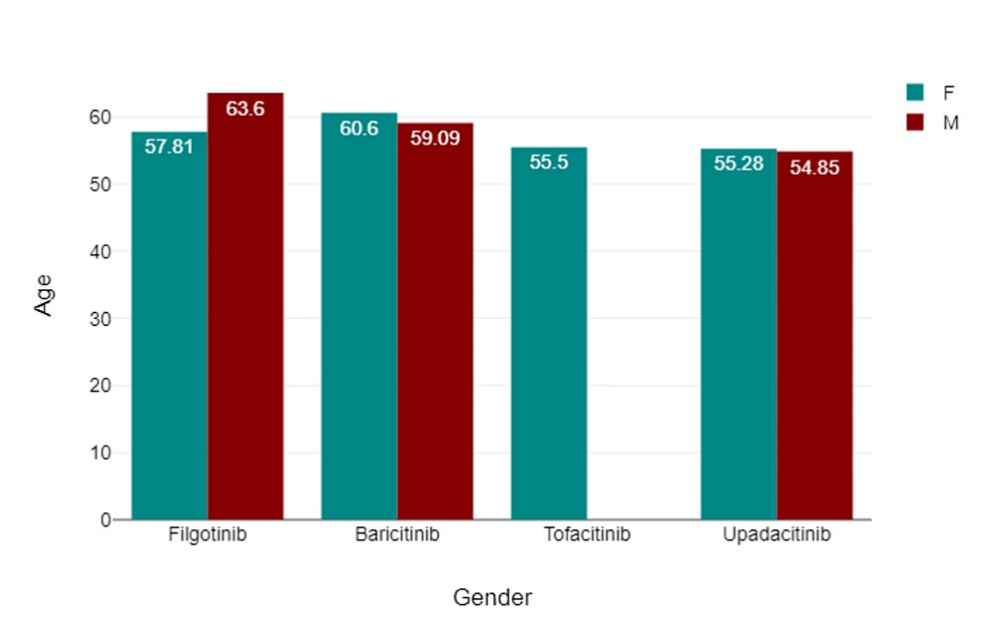
Mean age (in years) of patients and type of JAK inhibitor used by gender

**Figure 5:**
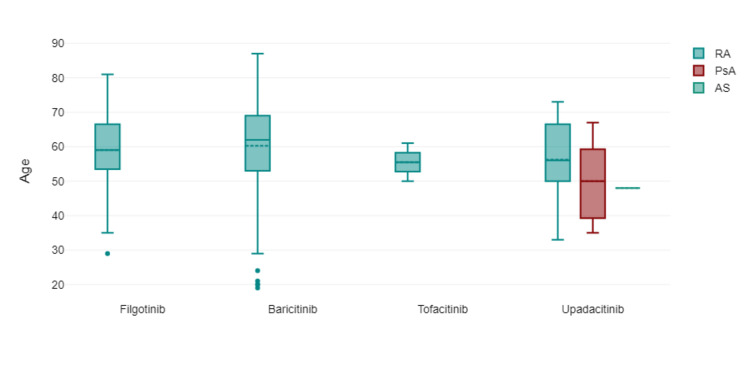
Type of JAK inhibitor by rheumatic disease sub-type Abbreviations: RA=rheumatoid arthritis; PsA=psoriatic arthritis; AS=ankylosing spondylitis

Figure 2 shows the age distribution of the patients via box plot by type of JAK inhibitor used. Figure 3 shows via box plot that there was no numerical or statistical significance in the number of cardiovascular events via a type of JAK inhibitor therapy. Figures 4-5 show both gender and age with disease subtype distribution by JAK inhibitors respectively. 

The study population was predominantly from a deprived area with a deprivation decile of 3 (1=most deprived; 10=least deprived), with the majority (13%) of participants belonging to this deprived deprivation decile. Around 7% of the participants were from the most deprived areas (deprivation decile 1) and 10.2% were from the least deprived areas (deprivation decile 10).

Inferential statistics: Use of the age of the patient and deprivation decile as independent variables

The multivariate logistic regression analysis was performed to determine the independent predictors (age and deprivation decile) for cardiovascular events while adjusting for potential confounders, including gender, ethnicity, disease duration, and co-morbidities. Tables 3-4 show that the regression model and model summary were statistically significant. 

**Table 3:** Chi-square value for the logistic regression model

Chi^2^	Degrees of freedom	p-value
24.04	13	0.031

**Table 4:** Model summary values for the logistic regression model

-2 Log-Likelihood	Cox & Snell R2	Nagelkerke R2	McFadden’s R2
95.98	0.07	0.23	0.2

The age of the patient and deprivation decile (independent variables) in the regression model highlighted the risk of cardiovascular events in patients on the various JAK inhibitor therapies. The results revealed in Table 5 show a statistically significant model using both the age of the patient and the deprivation decile together.

**Table 5:** P-value, odds ratio, and 95% confidence interval of the independent variables.

	Coefficient B	Standard error	z-Value	p-Value	Odds Ratio	95% conf. interval
Constant	-6.89	2.24	3.08	0.002	0	0 - 0.08
Biologic Baricitinib	0.35	0.83	0.42	0.675	1.41	0.28 - 7.17
Biologic Tofacitinib	-18.93	51994.71	0	1	0	Unbounded
Biologic Upadacitinib	0.02	1.3	0.02	0.987	1.02	0.08 - 12.95
Age and Deprivation Decile	0.06	0.03	2.05	0.04	1.06	1 - 1.13
Deprivation Decile 3	0.43	0.97	0.45	0.654	1.54	0.23 - 10.25
Deprivation Decile 8	0.05	1.06	0.05	0.962	1.05	0.13 - 8.39
Deprivation Decile 7	0.75	0.89	0.83	0.404	2.11	0.37 - 12.15
Deprivation Decile 2	1.24	1.1	1.13	0.259	3.47	0.4 - 30.12
Deprivation Decile 6	-0.6	1.28	0.47	0.64	0.55	0.04 - 6.76

**Figure 6:**
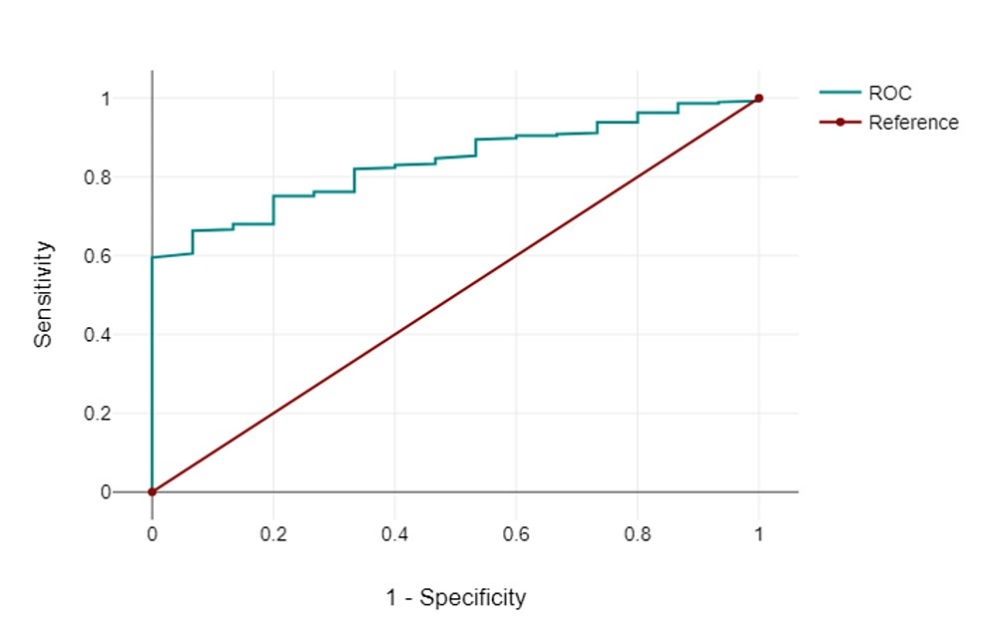
ROC Curve of the logistic regression model

Figure 6 shows the ROC curve of the logistic regression model.

Higher Q-risk (QRISK®3) scores were positively correlated with higher deprivation deciles, indicating a greater cardiovascular risk in patients from more deprived areas. The logistic regression analysis showed that the model as a whole was significant (Chi2(14) = 24.04, p 0.031, n = 309). The results showed that both the age of the patient and deprivation decile (OR: 1.06., 95% CI: 1.00-1.13, p = 0.04) together were significant predictors of cardiovascular events when using JAK inhibitor therapy. No difference was found in either the age of the patient or the deprivation decile alone. The coefficient of the variable, the age of the patient is b = 0.06, which is positive. The p-value of 0.04 indicates that this influence is statistically significant. The area under the curve (AUC) of the ROC curve was 0.837.

Statistical analysis was also performed to examine the influence of any of the JAK inhibitor therapies on cardiovascular risk, i.e. either baricitinib, filgotinib, tofacitinib, or upadacitinib. None were identified in this cohort to predict or have an influence on cardiovascular events. The coefficient of the variables for various JAK inhibitors was therefore not statistically significant. The coefficient of the independent variable for the deprivation decile from 1 to 10 was also not statistically significant.

## Discussion

This retrospective study evaluated the predictive value of the age of the patient and deprivation decile on cardiovascular events in patients using JAK inhibitor therapies. We observed a statistically significant association (p-value, 0.04) when considering the deprivation decile and the age of the patient together, highlighting the importance of incorporating socioeconomic factors for a comprehensive understanding of cardiovascular risk. The use of any JAK inhibitor therapy along with the age of the patient alone did not show any influence on cardiovascular events from this cohort. The majority of the patients were on baricitinib (70%). 

Our findings have several important implications for clinical practice. First, they suggest that the age of the patient alone may not be sufficient to predict cardiovascular risk in patients on JAK inhibitors. Second, they emphasize the importance of considering non-clinical factors such as socioeconomic factors (in this case deprivation decile) in cardiovascular risk assessment for this patient population on JAK inhibitor therapy. Socioeconomic factors can have a significant impact on healthcare outcomes, including cardiovascular risk. Patients from more deprived areas may face challenges such as limited access to healthcare resources, higher prevalence of comorbidities, and suboptimal management of cardiovascular risk factors [13]. By considering the deprivation decile in this regression model, the authors were able to identify a joint association between the age of the patient and cardiovascular events beyond traditional risk factors alone.

Interestingly, while Q-risk (QRISK®3) has been widely used as a validated tool for estimating cardiovascular risk, we found that it did not alone demonstrate a significant prediction for cardiovascular events when analyzed in isolation for patients using JAK inhibitor therapy. This suggests that Q-risk (QRISK®3) alone may not capture the full complexity of cardiovascular risk in patients with autoimmune disease using JAK inhibitor therapy [14]. These findings therefore emphasize the importance of considering additional factors, such as deprivation decile, to enhance the accuracy of risk prediction models. By accounting for socioeconomic status, healthcare providers can better identify patients who may be at a higher risk of cardiovascular events and implement preventive measures to mitigate these risks [15].

It is worth noting again that specific JAK inhibitor therapies alone are not enough to conclude that patients are at risk of cardiovascular events from this study cohort. These results support previous research indicating JAK inhibitors overall may not confer a higher cardiovascular risk compared to other therapies [4,16,17,18]. It is essential therefore for healthcare providers to be aware of this in order to tailor and optimize treatment decisions and closely monitor patients who may be at increased risk without withholding valuable therapies when needed [19,20].

Strengths and limitations of the study

The strength of the study is the large number of patients on JAK inhibitor therapy (N=309) included in the study from a diverse ethnic population of Caucasian, South Asian, and Black patients, reflecting the wider United Kingdom population. Furthermore, the patient catchment for the three hospitals is over one million. However, this study is however limited by its retrospective design. Additionally, although the study was conducted across three hospital sites, all three hospitals belong to one Tertiary NHS University Hospitals Trust in a single country, which may limit its generalizability to other populations of other countries.

Interpretation

This study provides evidence that age and deprivation decile in combination are independent risk factors for cardiovascular events in patients treated with JAK inhibitors. Clinicians should be aware of these risk factors and monitor patients closely for cardiovascular events.

Generalisability

The results of this study may be generalizable to other populations of patients with rheumatoid arthritis, psoriatic arthritis, or ankylosing spondylitis who are treated with JAK inhibitors. However, as the study was conducted in a single country, additional research is needed to confirm the findings in other populations.

## Conclusions

This study highlights the importance of considering both clinical and non-clinical factors, such as the age of the patient and deprivation decile, in predicting cardiovascular events in patients with autoimmune rheumatic diseases using JAK inhibitor therapies. Integrating socioeconomic factors into risk assessment models can improve the accuracy of cardiovascular risk prediction and enable healthcare providers to implement targeted preventive strategies. These findings contribute to the advancement of personalized medicine and the optimization of cardiovascular risk management in patients with rheumatic diseases.

Future studies should assess the exact age of the patient and deprivation decile values that have the maximum utility in predicting cardiovascular risk in this patient population. This would inform the development of more robust risk prediction models and facilitate the implementation of personalized preventive care strategies. Additionally, prospective studies with larger, more diverse cohorts are needed to validate these results and provide further clarity on the use of this model in clinical practice.

Overall, this study provides valuable insights into the importance of considering socioeconomic factors in cardiovascular risk assessment for patients using JAK inhibitor therapies. These findings have the potential to improve healthcare outcomes in patients with autoimmune rheumatic diseases.
